# Temporal Variations among Invasive Pneumococcal Disease Serotypes in Children and Adults in Germany (1992–2008)

**DOI:** 10.1155/2010/874189

**Published:** 2010-06-30

**Authors:** Matthias Imöhl, Ralf René Reinert, Mark van der Linden

**Affiliations:** ^1^National Reference Center for Streptococci, Institute of Medical Microbiology, University Hospital (RWTH), 52074 Aachen, Germany; ^2^Wyeth Vaccines Research, Coeur Défense-Tour A, 110 esplanade du Général de Gaulle, 92931 Paris la Défense Cedex, France

## Abstract

Nationwide surveillance of invasive pneumococcal disease has been conducted in Germany since 1992. From 1992 to 2008, a total of 12,137 isolates from invasive pneumococcal disease were collected. Data on serotypes were available for 9,394 invasive isolates. The leading serotypes were serotypes 14 (16.5%), 3 (8.0%), 7F (7.6%), 1 (7.3%), and 23F (6.0%). Variations in serotype distribution over the years are particularly extensive, especially concerning serotype 14 (min 7.4%, max 33.5%) with the highest percentages among the isolates serotyped from around 1997 to 2006. Serotypes 1 and 7F increased over the last decade. No increase was observed concerning serotype 19A. Higher pneumococcal conjugate vaccine coverages were observed among children (7v, 57.3%; 10v, 72.8%; 13v, 83.5%) than among adults (7v, 39.9%; 10v, 55.5%; 13v, 73.5%). The temporal variations in serotype distribution have to be kept in mind when interpreting vaccine coverages reported in epidemiological studies.

## 1. Introduction

*Streptococcus pneumoniae* is one of the most important pathogens in bacterial pneumonia, sepsis, and meningitis worldwide [1]. Significant temporal [2–4] and regional [5–7] variations among pneumococcal serotypes have been described. While the “epidemic” serogroups (serogroups 1-3 and 5) decreased considerably in the United States during the last century, the serogroups included in the seven-valent pneumococcal conjugate vaccine (PCV7) increased clearly [2]. A similar trend has been described in Spain, where the prevalence of PCV7 serotypes increased significantly (except serotype 4) since the start of the study in the early 1980s but then decreased considerably in the 2000s for all PCV7 serotypes except serotype 23F. Among the “epidemic” serotypes, a significant decrease of serotypes 1 and 5 has been observed in the 1980s followed by a significant increase in the late 1990s, while serotype 3 decreased continuously during the observational period. Furthermore, serotypes 6A, 7F, and 19A increased significantly since the late 1990s [4]. 

The widespread use of antibiotics and the increasing application of pneumococcal conjugate vaccines (a general recommendation of pneumococcal conjugate vaccination for children <2 years in Germany was issued at the end of July 2006) will have an impact on future changes in serotype distribution. 

The NRCS has conducted surveillance for invasive pneumococcal disease in Germany since 1992 [8]. Between 1992 and 1996 surveillance was based on a limited number of laboratories in Germany on a voluntary basis [9]. In 1996, nationwide population-based surveillance of IPD in children was started [10]. This surveillance system was extended to adults in one federal state (North-Rhine Westphalia) in 2001 [11] and successively broadened to nationwide surveillance until 2009. 

The aim of this study was to evaluate the serotype distribution of *S. pneumoniae* among the isolates with invasive pneumococcal disease (IPD) that were sent to the German National Reference Center for Streptococci (NRCS) between 1992 and 2008, analyze temporal trends, and calculate the vaccine coverages for the 7-valent, 10-valent, and 13-valent pneumococcal conjugate vaccines. 

## 2. Materials and Methods

### 2.1. Study Design

In this study data about invasive disease caused by *Streptococcus pneumoniae* in children (<16 years) and adults (≥16 years) in Germany were included using the data sources described above. Cases from January 1, 1992 to December 31, 2008 were included in this study. A case of IPD was defined by the isolation of *S. pneumoniae* from a normally sterile site.

### 2.2. Microbiological Investigations

Isolates were identified using standard procedures including bile solubility and optochin sensitivity. As a control strain, *Streptococcus pneumoniae* ATCC 49619 was used. Pneumococcal isolates were serotyped by the Neufeld's Quellung reaction using type- and factor-specific antisera (Statens Serum Institut, Copenhagen, Denmark). Among isolates of adults, high levels of resistance were a main trigger for initiation of serotyping during the early years of this study. Since cross-reactive serotypes were not included in the calculation, coverage information strictly refers to the serotypes included in the vaccines. Serogroup 6 isolates were not tested with respect to serotype 6C.

## 3. Results

**Figure 1:**
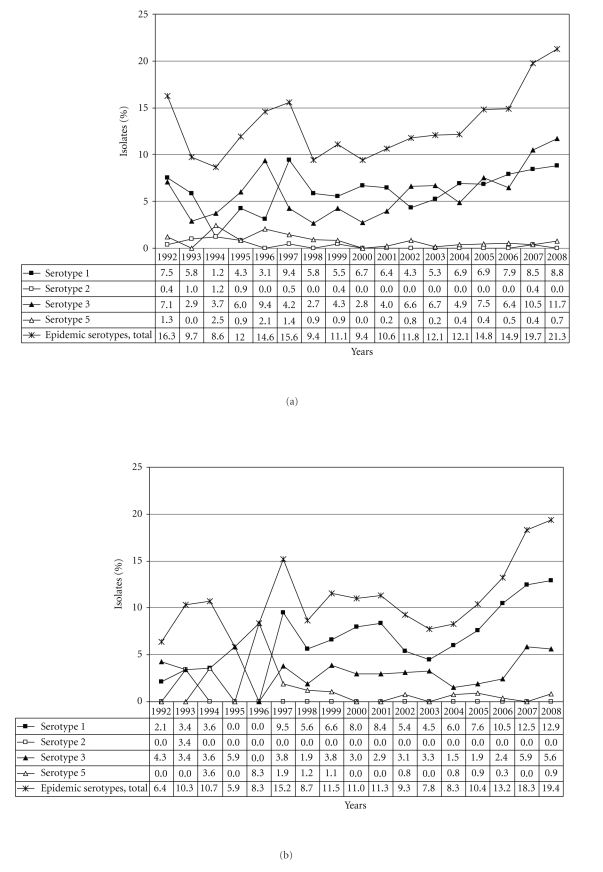

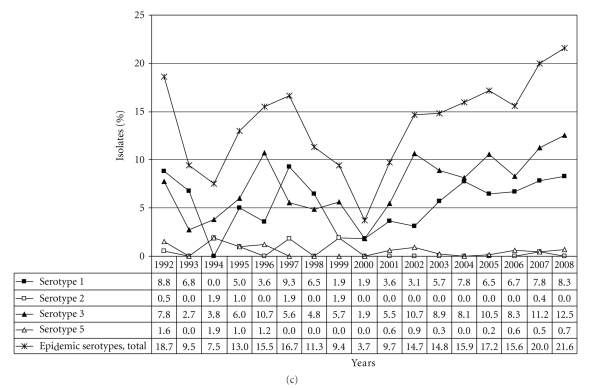
(a) Distribution of the epidemic serotypes 1, 2, 3, and 5 in percent (1992–2008, *n* = 9,394) in all age groups. (b) Distribution of the epidemic serotypes 1–3 and 5 among children in percent (1992–2008, *n* = 2,948). (c) Distribution of the epidemic serotypes 1–3 and 5 among adults in percent (1992–2008, *n* = 6,446).

**Figure 2:**
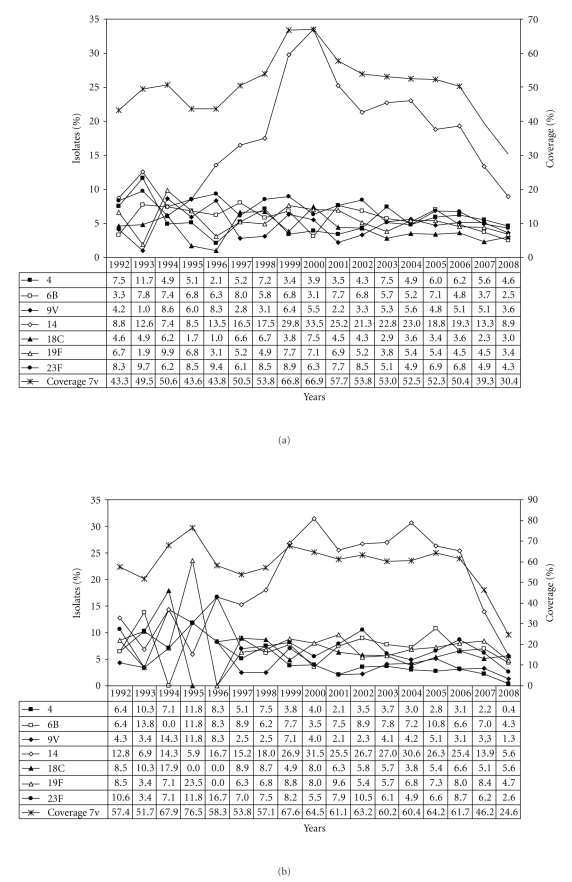

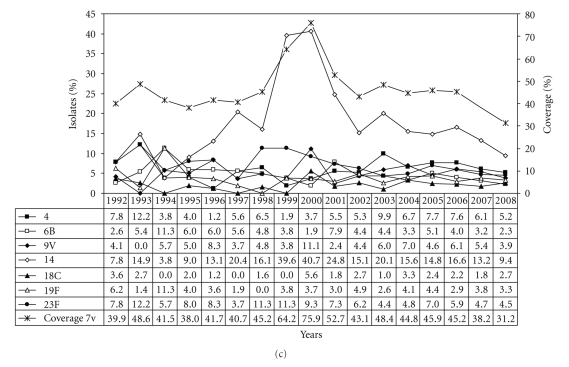
(a) Distribution of the 7-valent pneumococcal conjugate vaccine serotypes and the 7-valent vaccine coverage in percent (1992–2008, *n* = 9,394). (b) Distribution of the 7-valent pneumococcal conjugate vaccine serotypes and the 7-valent vaccine coverage among children in percent (1992–2008, *n* = 2,948). (c) Distribution of the 7-valent pneumococcal conjugate vaccine serotypes and the 7-valent vaccine coverage among adults in percent (1992–2008, *n* = 6,446).

A total of 12,137 isolates from invasive pneumococcal disease were collected between January 1, 1992 and December 31, 2008. The total numbers of cases for each year varied between 297 and 2,037 cases (median: 505 cases). Data on serotypes were available for 9,394 isolates (77.4% of all invasive isolates; 31.4% children, 68.6% adults; 54.9% male, 43.7% female, 1.4% no information on gender). The serotype distribution of the years 1992–2008 is shown in Table 1(a). The leading serotypes were serotypes 14 (16.5%), 3 (8.0%), 7F (7.6%), 1 (7.3%), and 23F (6.0 %). Concerning the epidemic serotypes 1–3 and 5, a slight increase was noticed during the last years, reaching levels around 20% among children and adults in 2008 (Figures 1(a)–1(c)). Variations in serotype distribution over the years are partly extensive, especially concerning serotype 14 (min 7.4%, max 33.5%) (Figure 2(a)). Differences in serotype distribution among children and adults are shown in Tables 1(b) and 1(c). The amount of serotyped isolates among all pneumococcal isolates sent to the NRCS in Germany is shown in Table 2. Over the years the percentage of isolates serotyped increased continuously and in the last four years of the study almost all isolates were serotyped. 

**Figure 3:**
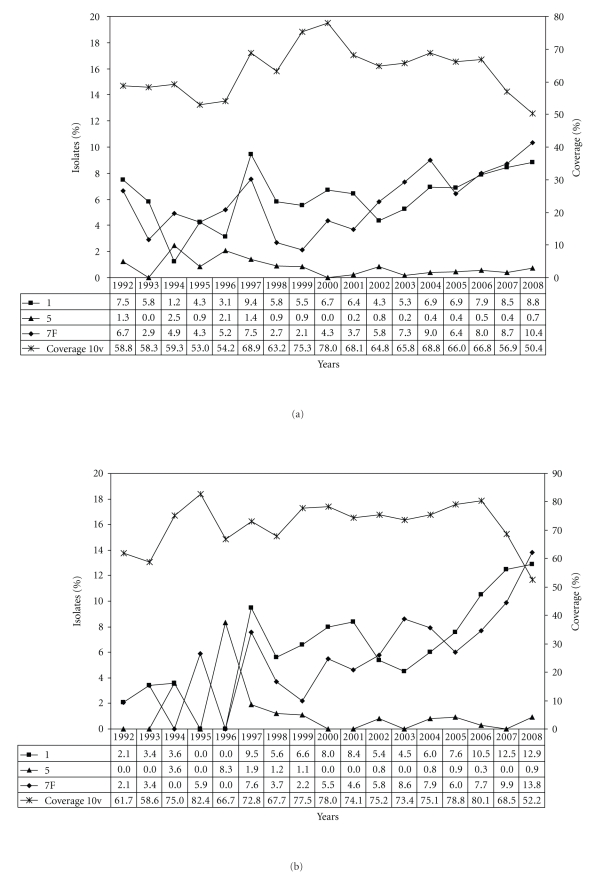

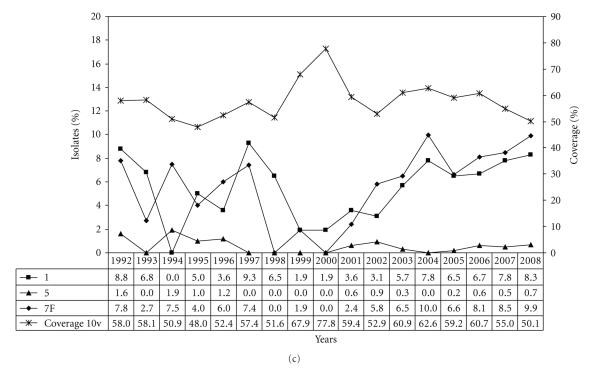
(a) Distribution of the additional 10v-pneumococcal conjugate vaccine serotypes 1, 5, and 7F and the 10-valent vaccine coverage in percent (1992–2008, *n* = 9,394). (b) Distribution of the additional 10v-pneumococcal conjugate vaccine serotypes 1, 5, and 7F and the 10-valent vaccine coverage among children in percent (1992–2008, *n* = 2,948). (c) Distribution of the additional 10v-pneumococcal conjugate vaccine serotypes 1, 5, and 7F and the 10-valent vaccine coverage among adults in percent (1992–2008, *n* = 6,446).

**Figure 4:**
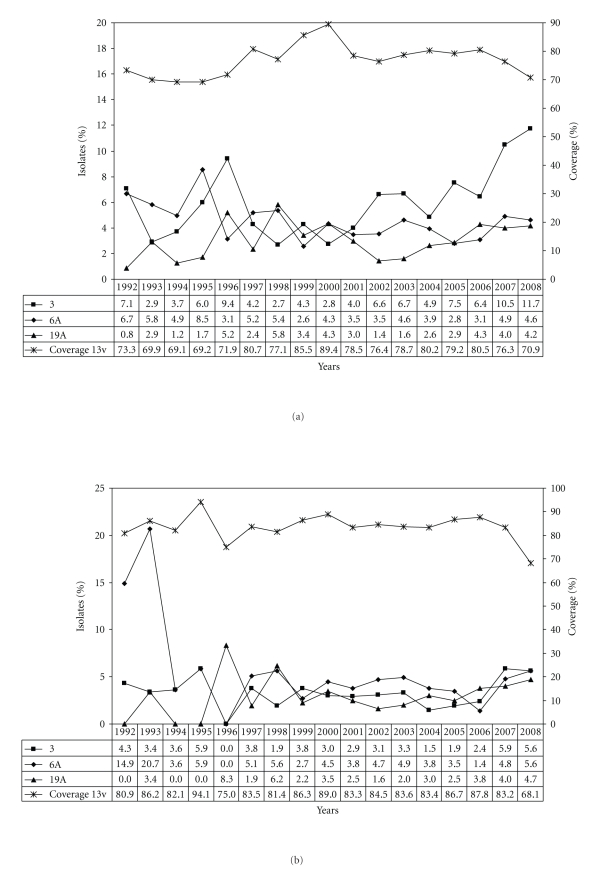

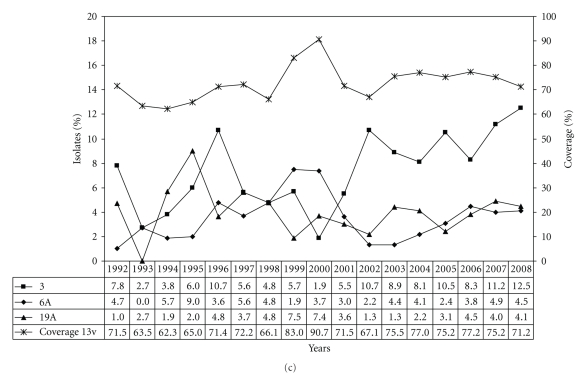
(a) Distribution of the additional 13v-pneumococcal conjugate vaccine serotypes 3, 6A, and 19A and the 13-valent vaccine coverage in percent (1992–2008, *n* = 9,394). (b) Distribution of the additional 13v-pneumococcal conjugate vaccine serotypes 3, 6A, and 19A and the 13-valent vaccine coverage among children in percent (1992–2008, *n* = 2,948). (c) Distribution of the additional 13v-pneumococcal conjugate vaccine serotypes 3, 6A, and 19A and the 13-valent vaccine coverage among adults in percent (1992–2008, *n* = 6,446).

Serotype 14 is considerably more frequent among children (22.5%) than among adults (13.7%). Overall, an increase of serotype 14 can be noticed from around 1997 to 2006 (Figure 2(a)). A high percentage of serotype 14 isolates remain in existence for a longer time among children (1999–2006, Figure 2(b)) than among adults (1999–2001, Figure 2(c)). Serotype 1 and serotype 7F have been clearly increasing over the last approximately 10 years, both among children and among adults. While for children the percentage of serotypes 1 and 7F reach the highest values during this study in 2008, among adults they are high, but still within the range already noted before (Figures 3(a)-3(c)). Serotype 3 is far more common among adults (10.2%) than among children (3.3%) (Tables 1(b)-1(c)). The increase observed during the last years seems to be slightly higher among adults than among children (Figures 4(a)–4(c)). Concerning serotype 19A no clear change in frequency can be observed during the period under study (Figures 4(a)–4(c)). 

**Figure 5:**
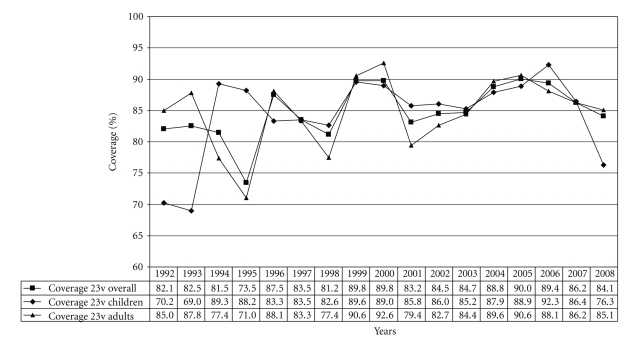
Coverage of the 23v-pneumococcal polysaccharide vaccine for children (*n* = 2,948), adults (*n* = 6,446), and overall (*n* = 9,394).

Variations in serotype distribution also affect theoretical vaccine coverages. The overall serotype coverage for the 7-valent conjugate vaccine was 45.4% during 1992–2008. For the 10-valent vaccine and the 13-valent vaccine the overall coverages were 60.9% and 76.6%, respectively. Generally, higher coverages were observed among children (7v, 57.3%; 10v, 72.8%; 13v, 83.5%) than among adults (7v, 39.9%; 10v, 55.5%; 13v, 73.5%). Among children, the coverage of the 7-valent vaccine decreased from 64.2% in 2005 to 24.6% in 2008 (Figure 2(b)), while for adults the coverage declined to a lesser extent (2005, 45.9%; 2008, 31.2%) (Figure 2(c)). Temporal changes of the additional 10-valent and 13-valent pneumococcal vaccine serotypes and the corresponding vaccine coverages are shown in Figures 3(a)–3(c) (10v) and Figures 4(a)–4(c) (13v). Since the general recommendation of pneumococcal conjugate vaccination for children <2 years in Germany at the end of July 2006 a percentage increase among the new (10v) or upcoming (13v) vaccine serotypes was noticed especially for serotypes 1 and 7F in children (Figure 3(b)) and for serotype 3 in adults (Figure 4(c)). In comparison, coverages of the 23-valent polysaccharide vaccine are very similar among children (min, 69.0%; max, 92.3%) and adults (min, 71.0%; max, 92.6%) from 1992 to 2008 (Figure 5).

## 4. Discussion

In this paper we present the results of 17 years of surveillance concerning serotypes of invasive pneumococcal disease in Germany. 

In the present study serotype 14 was the most prevalent serotype among children, followed in frequency by the serotypes 1, 6B, 19F, 23F, and 7F, respectively. These data are similar to those published for German children previously [12, 13] and in line with data from England [14], Belgium [15], and Denmark [16]. Among adults, the most frequent serotypes were 14, 3, 7F, 1, 4, and 23F (sorted in descending order). These results are similar to a previous study from the NRCS among adults in North-Rhine Westphalia, Germany, between 2003 and 2006 [17], and are generally in line with results reported from other countries [6, 15, 18–20]; however, they deviate in part from older German data [12, 21]. 

The variation of serotype 14 over the years is extensive in our study with an increased prevalence from about 1997 to 2006, reaching maximum values in 2000. Although the rate of serotyped isolates among adults was low from 1999 to 2001 (1999, 22.9%; 2000, 26.6%; 2001, 38.6%), the increase seems plausible. First, the increase among adults is paralleled by an increase among children, where nearly all isolates have been serotyped. Second, the rate of isolates serotyped among adults is within the range serotyped in the years before 1999, where a similar increase of serotype 14 was not observed. Furthermore, similar data have been shown in a report from Spain [4]. Also, a small rise in frequency of serotype 14 has been reported from Denmark in 1995–1999 [16], and data from England demonstrated a rise in incidence in 2000 and 2001 [14]. The rise of serotypes 1 and 7F for nearly one decade is similar to results from Spain [4]. 

Concerning serotype 19A, the highest prevalences were observed in 1996 (8.3%) and 1998 (6.2%) among children, and in 1999 (7.5%) and 2000 (7.4%) among adults. Although there seems to be a slight upward movement during the years 2002 to 2008, the prevalences still are below the maximum values reported. The increase of serotype 19A reported from other countries [4, 22, 23] has not been found in Germany so far. 

Although changes in the incidence of different serotypes have been reported, the reasons for this are not fully understood [14]. Potential reasons discussed are changes in socioeconomic conditions, antibiotic use and resistance levels, immunocompromised status of populations, precise age distributions of the populations studied and increased life expectancy, and blood-culturing rates [2, 6, 24]. Since the general recommendation of pneumococcal conjugate vaccination for children <2 years in Germany at end of July 2006 a reduction in the percentage of IPD caused by the 7-valent vaccine serotypes was observed [25]. This effect is more apparent among children, but also present among the adult population. Similar results have been reported from other countries [3, 4, 26–29]. Logically, the percentage decrease in vaccine serotypes is paralleled by a percentage increase in the amount of IPD caused by nonvaccine serotypes, which was most prominent for serotypes 1 and 7F in children and serotype 3 in adults in this study. Serotype coverage for the 7-valent conjugate vaccine in Germany was 45.4% from 1992 to 2008. Calculated coverages for the 10-valent vaccine and 13-valent vaccine are 60.9% and 76.6%, respectively. These data are comparable to those previously reported [5, 14, 30, 31]. Coverages of all three vaccine formulations in our study are approximately 15% (7v, 17.4%; 10v, 17.3%; and 13v, 10.0%) higher among children than among adults. Differences in age distribution among study populations are known to have a major impact on vaccine coverage of the current and proposed vaccines for different age groups and, furthermore, comparable data have been published [14]. 

Nevertheless, some factors and limitations must be regarded when interpreting the results of this study. First of all, the isolates were sent by the participating laboratories on a voluntary basis, as participation in surveillance is not mandatory in Germany. Furthermore, the systematic sampling of invasive isolates from adults (1992) and children (1997) was taken up at different points of time, and the included population-based studies in three German federal states started in 2001 (North Rhine-Wesphalia) and 2006 (Bavaria and Saxony). Among isolates of adults, high levels of resistance were a main trigger for initiation of serotyping during the early years of this study. Therefore, the isolates may not be fully representative of all IPD in Germany over the last two decades. 

Moreover, the structure of the surveillance project has been continousely improved over the 15 years and, in particular, after the general recommendation of pneumococcal conjugate vaccine for children <2 years in Germany at the end of July 2006 an increased disease awareness by both clinical micobiologists and pediatricians was observed [32]. 

Ongoing nationwide surveillance is necessary to observe further developments of pneumococcal serotype distribution in Germany.

## Figures and Tables

**Table:** (a) Serotype distribution of IPD in Germany (1992–2008, *n* = 9,394) in all age groups.

Sero- type	1992 (%)	1993 (%)	1994 (%)	1995 (%)	1996 (%)	1997 (%)	1998 (%)	1999 (%)	2000 (%)	2001 (%)	2002 (%)	2003 (%)	2004 (%)	2005 (%)	2006 (%)	2007 (%)	2008 (%)	Total (%)	Total (*n*)
14	8.8	12.6	7.4	8.5	13.5	16.5	17.5	29.8	33.5	25.2	21.3	22.8	23.0	18.8	19.3	13.3	8.9	16.5	1,549
3	7.1	2.9	3.7	6.0	9.4	4.2	2.7	4.3	2.8	4.0	6.6	6.7	4.9	7.5	6.4	10.5	11.7	8.0	755
7F	6.7	2.9	4.9	4.3	5.2	7.5	2.7	2.1	4.3	3.7	5.8	7.3	9.0	6.4	8.0	8.7	10.4	7.6	718
1	7.5	5.8	1.2	4.3	3.1	9.4	5.8	5.5	6.7	6.4	4.3	5.3	6.9	6.9	7.9	8.5	8.8	7.3	690
23F	8.3	9.7	6.2	8.5	9.4	6.1	8.5	8.9	6.3	7.7	8.5	5.1	4.9	6.9	6.8	4.9	4.3	6.0	559
4	7.5	11.7	4.9	5.1	2.1	5.2	7.2	3.4	3.9	3.5	4.3	7.5	4.9	6.0	6.2	5.6	4.6	5.4	507
6B	3.3	7.8	7.4	6.8	6.3	8.0	5.8	6.8	3.1	7.7	6.8	5.7	5.2	7.1	4.8	3.7	2.5	4.8	449
19F	6.7	1.9	9.9	6.8	3.1	5.2	4.9	7.7	7.1	6.9	5.2	3.8	5.4	5.4	4.5	4.5	3.4	4.8	447
9V	4.2	1.0	8.6	6.0	8.3	2.8	3.1	6.4	5.5	2.2	3.3	5.3	5.6	4.8	5.1	5.1	3.6	4.5	425
6A	6.7	5.8	4.9	8.5	3.1	5.2	5.4	2.6	4.3	3.5	3.5	4.6	3.9	2.8	3.1	4.9	4.6	4.3	401
18C	4.6	4.9	6.2	1.7	1.0	6.6	6.7	3.8	7.5	4.5	4.3	2.9	3.6	3.4	3.6	2.3	3.0	3.5	326
19A	0.8	2.9	1.2	1.7	5.2	2.4	5.8	3.4	4.3	3.0	1.4	1.6	2.6	2.9	4.3	4.0	4.2	3.4	320
22F	1.3	2.9	3.7	0.0	2.1	0.5	0.9	0.0	0.0	0.7	1.0	1.0	1.5	3.0	3.0	2.9	3.0	2.2	208
8	2.1	1.9	0.0	0.9	2.1	0.5	0.9	0.0	0.4	1.5	2.3	2.1	2.1	2.5	2.2	2.5	3.0	2.2	208
10A	1.7	1.0	3.7	2.6	2.1	1.4	1.3	0.9	1.6	1.7	2.5	0.6	2.2	1.5	1.9	1.9	2.2	1.8	172
9N	2.1	1.0	1.2	3.4	1.0	1.4	0.9	1.3	1.6	1.2	1.7	1.3	1.3	1.4	1.6	2.0	2.6	1.8	172
11A	2.5	1.9	1.2	1.7	4.2	1.4	0.4	0.9	0.0	0.2	0.6	1.8	1.5	1.3	1.1	1.7	2.5	1.6	149
12F	2.5	1.9	2.5	1.7	6.3	1.4	1.3	1.3	0.0	2.2	1.0	1.8	0.7	0.4	0.4	1.0	1.7	1.3	118
24F	2.1	1.0	1.2	0.9	1.0	0.5	1.8	1.3	0.8	2.0	0.6	1.3	1.5	0.7	1.2	0.9	1.8	1.2	116
23A	0.8	1.0	1.2	0.0	0.0	0.9	1.3	0.0	0.8	0.5	0.8	0.8	0.2	0.4	0.8	1.0	1.7	0.9	88
15B	0.8	1.9	0.0	0.0	1.0	0.0	0.9	0.9	1.2	0.0	0.6	0.3	0.9	1.1	0.9	1.0	1.2	0.9	83
35F	0.8	0.0	1.2	0.9	0.0	0.5	0.4	0.9	0.8	0.5	0.0	0.3	0.0	0.4	0.5	0.7	1.8	0.8	74
33F	1.3	1.0	1.2	0.9	0.0	0.9	1.8	0.9	0.0	0.5	0.6	1.0	0.9	0.7	0.2	0.6	0.9	0.7	68
38	0.4	0.0	2.5	0.0	0.0	0.9	0.4	0.0	0.4	0.5	0.6	0.6	0.7	0.2	0.3	0.4	1.4	0.6	61
5	1.3	0.0	2.5	0.9	2.1	1.4	0.9	0.9	0.0	0.2	0.8	0.2	0.4	0.4	0.5	0.4	0.7	0.6	55
15C	1.7	0.0	0.0	1.7	0.0	3.8	0.0	0.0	0.4	0.0	0.4	0.6	0.4	0.6	0.9	0.5	0.3	0.6	53
15A	0.0	0.0	0.0	1.7	0.0	0.0	1.8	1.7	0.0	0.7	1.2	0.8	0.7	0.6	0.5	0.1	0.4	0.5	48
9A	0.4	1.0	1.2	0.9	2.1	0.9	0.9	0.0	0.8	3.5	2.1	0.6	0.0	0.4	0.0	0.2	0.0	0.5	47
20	0.8	1.9	2.5	0.0	0.0	0.0	0.0	0.4	0.0	0.0	0.0	0.6	0.6	0.8	0.3	0.6	0.4	0.5	43
NT	0.0	0.0	0.0	0.0	1.0	0.0	0.0	0.4	0.0	0.2	0.2	0.0	0.7	0.4	0.3	0.7	0.5	0.4	40
17F	0.0	1.9	0.0	0.9	0.0	0.0	0.9	0.0	0.0	0.0	1.2	0.3	0.7	0.8	0.3	0.4	0.2	0.4	39
16F	0.0	0.0	0.0	1.7	1.0	0.5	0.0	0.4	0.0	1.0	0.6	0.5	0.4	0.2	0.4	0.1	0.4	0.4	34
33A	0.0	0.0	0.0	0.0	0.0	0.0	0.0	0.0	0.0	0.0	0.0	0.0	0.4	0.8	0.8	0.5	0.2	0.3	30
31	0.8	0.0	1.2	0.9	0.0	0.0	0.0	0.0	0.0	0.5	0.4	0.5	0.0	0.1	0.3	0.1	0.5	0.3	26
18A	0.0	0.0	1.2	0.0	0.0	0.0	0.0	0.0	0.0	0.5	0.4	0.6	0.2	0.2	0.1	0.3	0.2	0.2	22
34	0.8	0.0	0.0	0.0	0.0	0.0	0.4	0.0	0.0	0.0	0.4	0.3	0.2	0.2	0.2	0.3	0.2	0.2	22
rough	0.0	1.0	1.2	0.9	1.0	0.0	0.0	0.0	0.4	0.5	1.0	1.3	0.0	0.0	0.0	0.0	0.0	0.2	20
23B	0.0	1.0	0.0	0.0	0.0	0.5	0.4	0.0	0.0	0.5	0.0	0.2	0.4	0.1	0.1	0.1	0.2	0.2	17
9L	0.0	0.0	0.0	0.0	0.0	0.0	0.4	0.0	0.0	0.7	0.8	0.3	0.2	0.0	0.2	0.2	0.0	0.2	17
13	0.0	2.9	0.0	0.0	0.0	0.9	0.0	0.0	0.0	0.2	0.6	0.0	0.2	0.0	0.0	0.1	0.1	0.2	15
18F	0.4	1.9	0.0	0.9	0.0	0.5	0.0	0.9	0.0	0.0	0.2	0.2	0.0	0.1	0.0	0.3	0.0	0.2	15
2	0.4	1.0	1.2	0.9	0.0	0.5	0.0	0.4	0.0	0.0	0.0	0.0	0.0	0.0	0.0	0.4	0.0	0.1	13
12A	0.0	0.0	0.0	0.0	0.0	0.5	0.0	0.4	0.0	0.0	0.2	0.0	0.2	0.0	0.3	0.2	0.0	0.1	12
28A	0.0	0.0	0.0	0.0	0.0	0.5	0.0	0.0	0.4	0.0	0.4	0.2	0.0	0.1	0.1	0.2	0.0	0.1	12
35B	0.0	0.0	0.0	0.0	0.0	0.0	0.0	0.0	0.0	0.0	0.2	0.0	0.2	0.0	0.0	0.2	0.2	0.1	11
10B	0.0	0.0	0.0	0.0	0.0	0.0	0.4	0.0	0.0	0.0	0.0	0.0	0.2	0.2	0.0	0.2	0.1	0.1	10
Others^⋆^	2.9	1.9	2.5	7.7	3.1	0.5	4.9	1.7	1.2	1.5	0.6	1.6	0.6	1.3	0.3	1.7	0.9	1.4	130
Total	100	100	100	100	100	100	100	100	100	100	100	100	100	100	100	100	100	100	9,394

Not sero- typed	52.5	77.8	76.0	67.0	67.7	40.8	40.5	43.9	38.2	39.4	29.9	21.5	19.1	1.5	0.0	0.1	0.0	22.6	2,743

NT: nontypeable; *n*: number of isolates tested.

Others^⋆^ includes the serotypes (number of isolates): 15F (9), 18B (9), 12B (8), 7C (8), 33B (7), 10F (6), 11B (6), 11F (6), 35A (6), 19C (5), 29 (5), 37 (5), 21 (4), 24A (4), 28F (4), 35C (4), 19B (3), 22A (3), 36 (3), 45 (3), 7A (3), 24B (2), 25F (2), 39 (2), 48 (2), 6 (2), 7B (2), 12 (1), 17A (1), 18 (1), 19 (1), 23 (1), 35 (1), 9 (1).

**Table:** (b) Serotype distribution of IPD in Germany (1992–2008, *n* = 2,948) in children (<16 years).

Sero-type	1992 (%)	1993 (%)	1994 (%)	1995 (%)	1996 (%)	1997 (%)	1998 (%)	1999 (%)	2000 (%)	2001 (%)	2002 (%)	2003 (%)	2004 (%)	2005 (%)	2006 (%)	2007 (%)	2008 (%)	Total (%)	Total (*n*)
14	12.8	6.9	14.3	5.9	16.7	15.2	18.0	26.9	31.5	25.5	26.7	27.0	30.6	26.3	25.4	13.9	5.6	22.5	664
1	2.1	3.4	3.6	0.0	0.0	9.5	5.6	6.6	8.0	8.4	5.4	4.5	6.0	7.6	10.5	12.5	12.9	7.9	234
6B	6.4	13.8	0.0	11.8	8.3	8.9	6.2	7.7	3.5	7.5	8.9	7.8	7.2	10.8	6.6	7.0	4.3	7.3	216
19F	8.5	3.4	7.1	23.5	0.0	6.3	6.8	8.8	8.0	9.6	5.4	5.7	6.8	7.3	8.0	8.4	4.7	7.2	213
23F	10.6	3.4	7.1	11.8	16.7	7.0	7.5	8.2	5.5	7.9	10.5	6.1	4.9	6.6	8.7	6.2	2.6	6.9	204
7F	2.1	3.4	0.0	5.9	0.0	7.6	3.7	2.2	5.5	4.6	5.8	8.6	7.9	6.0	7.7	9.9	13.8	6.9	204
18C	8.5	10.3	17.9	0.0	0.0	8.9	8.7	4.9	8.0	6.3	5.8	5.7	3.8	5.4	6.6	5.1	5.6	6.2	182
6A	14.9	20.7	3.6	5.9	0.0	5.1	5.6	2.7	4.5	3.8	4.7	4.9	3.8	3.5	1.4	4.8	5.6	4.4	130
9V	4.3	3.4	14.3	11.8	8.3	2.5	2.5	7.1	4.0	2.1	2.3	4.1	4.2	5.1	3.1	3.3	1.3	3.7	108
4	6.4	10.3	7.1	11.8	8.3	5.1	7.5	3.8	4.0	2.1	3.5	3.7	3.0	2.8	3.1	2.2	0.4	3.5	102
3	4.3	3.4	3.6	5.9	0.0	3.8	1.9	3.8	3.0	2.9	3.1	3.3	1.5	1.9	2.4	5.9	5.6	3.3	96
19A	0.0	3.4	0.0	0.0	8.3	1.9	6.2	2.2	3.5	2.5	1.6	2.0	3.0	2.5	3.8	4.0	4.7	3.1	90
10A	2.1	0.0	0.0	0.0	0.0	0.6	0.0	1.1	1.5	2.1	1.6	0.4	2.3	1.9	2.8	2.2	3.4	1.7	51
24F	4.3	3.4	0.0	0.0	0.0	0.0	2.5	1.6	1.0	2.1	0.8	2.5	1.9	0.6	1.4	1.1	2.2	1.5	44
15B	0.0	0.0	0.0	0.0	0.0	0.0	0.6	1.1	1.0	0.0	0.4	0.4	0.8	1.3	0.7	2.2	2.2	0.9	26
15C	2.1	0.0	0.0	0.0	0.0	4.4	0.0	0.0	0.0	0.0	0.4	0.8	0.4	1.3	1.4	0.4	2.2	0.9	26
9N	0.0	0.0	0.0	0.0	0.0	0.0	0.6	1.1	1.5	0.8	0.4	1.6	1.5	0.3	1.0	0.4	1.7	0.9	26
38	2.1	0.0	3.6	0.0	0.0	0.6	0.6	0.0	0.5	0.4	0.8	0.4	0.8	0.3	0.0	0.7	3.9	0.8	23
15A	0.0	0.0	0.0	0.0	0.0	0.0	1.2	1.6	0.0	0.4	1.2	1.6	0.8	0.6	1.0	0.0	0.4	0.7	21
22F	0.0	0.0	7.1	0.0	0.0	0.6	0.6	0.0	0.0	0.8	1.2	1.2	0.8	0.6	0.3	0.4	1.3	0.7	21
9A	0.0	0.0	0.0	0.0	0.0	1.3	0.6	0.0	1.0	3.8	1.9	0.8	0.0	0.0	0.0	0.0	0.0	0.7	21
5	0.0	0.0	3.6	0.0	8.3	1.9	1.2	1.1	0.0	0.0	0.8	0.0	0.8	0.9	0.3	0.0	0.9	0.6	19
8	0.0	0.0	0.0	0.0	0.0	0.6	1.2	0.0	0.5	0.4	1.2	0.4	0.8	0.9	0.7	0.4	0.4	0.6	18
33F	0.0	0.0	0.0	0.0	0.0	1.3	1.9	0.5	0.0	0.4	1.2	0.8	0.4	0.0	0.0	0.4	1.3	0.6	17
12F	2.1	0.0	3.6	0.0	0.0	0.0	0.6	1.1	0.0	1.7	0.0	0.0	0.8	0.0	0.0	0.4	1.7	0.5	16
11A	0.0	0.0	0.0	0.0	8.3	1.9	0.0	0.5	0.0	0.0	0.4	1.2	0.0	0.0	0.3	1.1	0.9	0.5	15
23A	2.1	0.0	0.0	0.0	0.0	0.6	1.2	0.0	0.5	0.0	0.4	0.4	0.0	0.0	0.7	0.4	1.7	0.5	14
18A	0.0	0.0	0.0	0.0	0.0	0.0	0.0	0.0	0.0	0.8	0.8	0.8	0.4	0.0	0.0	0.4	0.9	0.3	10
35F	0.0	0.0	0.0	0.0	0.0	0.0	0.6	0.5	1.0	0.0	0.0	0.0	0.0	0.3	0.3	0.4	1.3	0.3	10
NT	0.0	0.0	0.0	0.0	0.0	0.0	0.0	0.5	0.0	0.0	0.0	0.0	1.5	0.3	0.0	0.7	0.9	0.3	10
33A	0.0	0.0	0.0	0.0	0.0	0.0	0.0	0.0	0.0	0.0	0.0	0.0	0.4	1.3	0.7	0.4	0.4	0.3	9
rough	0.0	3.4	0.0	0.0	0.0	0.0	0.0	0.0	0.5	0.8	0.8	1.2	0.0	0.0	0.0	0.0	0.0	0.3	9
17F	0.0	0.0	0.0	0.0	0.0	0.0	1.2	0.0	0.0	0.0	0.0	0.0	0.8	0.3	0.0	0.4	0.9	0.3	8
16F	0.0	0.0	0.0	0.0	0.0	0.6	0.0	0.0	0.0	0.8	0.4	0.0	0.0	0.3	0.0	0.0	0.9	0.2	7
18B	0.0	0.0	0.0	0.0	8.3	0.0	1.2	0.5	0.5	0.0	0.0	0.0	0.0	0.6	0.0	0.0	0.0	0.2	7
23B	0.0	3.4	0.0	0.0	0.0	0.6	0.6	0.0	0.0	0.0	0.0	0.4	0.4	0.0	0.0	0.0	0.4	0.2	6
34	2.1	0.0	0.0	0.0	0.0	0.0	0.6	0.0	0.0	0.0	0.0	0.0	0.4	0.3	0.0	0.7	0.0	0.2	6
18F	0.0	0.0	0.0	0.0	0.0	0.6	0.0	1.1	0.0	0.0	0.4	0.0	0.0	0.0	0.0	0.4	0.0	0.2	5
20	0.0	0.0	0.0	0.0	0.0	0.0	0.0	0.5	0.0	0.0	0.0	0.4	0.4	0.3	0.0	0.4	0.0	0.2	5
9L	0.0	0.0	0.0	0.0	0.0	0.0	0.0	0.0	0.0	0.4	0.8	0.4	0.0	0.0	0.3	0.0	0.0	0.2	5
Others^⋆^	2.1	3.4	3.6	5.9	8.3	2.5	2.5	1.6	1.5	0.8	0.8	0.4	1.5	1.6	0.3	3.3	3.0	1.7	50
Total	100	100	100	100	100	100	100	100	100	100	100	100	100	100	100	100	100	100	2,948

Not sero- typed	7.8	17.1	3.4	34.6	61.3	7.1	2.4	3.2	3.8	0.0	0.4	0.8	0.0	0.0	0.0	0.0	0.0	2.4	72

NT: nontypeable; *n*: number of isolates tested.

Others^⋆^ includes the serotypes (number of isolates): 28A (4), 11B (3), 12A (3), 12B (3), 13 (3), 33B (3), 35B (3), 15F (2), 19C (2), 21 (2), 24A (2), 28F (2), 29 (2), 35A (2), 37 (2), 7A (2), 10F (1), 19B (1), 2 (1), 31 (1), 35 (1), 35C (1), 36 (1), 39 (1), 6 (1), 7C (1).

**Table:** (c) Serotype distribution of IPD in Germany (1992–2008, *n* = 6,446) in adults (≥16 years).

Sero-type	1992 (%)	1993 (%)	1994 (%)	1995 (%)	1996 (%)	1997 (%)	1998 (%)	1999 (%)	2000 (%)	2001 (%)	2002 (%)	2003 (%)	2004 (%)	2005 (%)	2006 (%)	2007 (%)	2008 (%)	Total (%)	Total (*n*)
14	7.8	14.9	3.8	9.0	13.1	20.4	16.1	39.6	40.7	24.8	15.1	20.1	15.6	14.8	16.6	13.2	9.4	13.7	885
3	7.8	2.7	3.8	6.0	10.7	5.6	4.8	5.7	1.9	5.5	10.7	8.9	8.1	10.5	8.3	11.2	12.5	10.2	659
7F	7.8	2.7	7.5	4.0	6.0	7.4	0.0	1.9	0.0	2.4	5.8	6.5	10.0	6.6	8.1	8.5	9.9	8.0	514
1	8.8	6.8	0.0	5.0	3.6	9.3	6.5	1.9	1.9	3.6	3.1	5.7	7.8	6.5	6.7	7.8	8.3	7.1	456
4	7.8	12.2	3.8	4.0	1.2	5.6	6.5	1.9	3.7	5.5	5.3	9.9	6.7	7.7	7.6	6.1	5.2	6.3	405
23F	7.8	12.2	5.7	8.0	8.3	3.7	11.3	11.3	9.3	7.3	6.2	4.4	4.8	7.0	5.9	4.7	4.5	5.5	355
9V	4.1	0.0	5.7	5.0	8.3	3.7	4.8	3.8	11.1	2.4	4.4	6.0	7.0	4.6	6.1	5.4	3.9	4.9	317
6A	4.7	0.0	5.7	9.0	3.6	5.6	4.8	1.9	3.7	3.0	2.2	4.4	4.1	2.4	3.8	4.9	4.5	4.2	271
19F	6.2	1.4	11.3	4.0	3.6	1.9	0.0	3.8	3.7	3.0	4.9	2.6	4.1	4.4	2.9	3.8	3.3	3.6	234
6B	2.6	5.4	11.3	6.0	6.0	5.6	4.8	3.8	1.9	7.9	4.4	4.4	3.3	5.1	4.0	3.2	2.3	3.6	233
19A	1.0	2.7	1.9	2.0	4.8	3.7	4.8	7.5	7.4	3.6	1.3	1.3	2.2	3.1	4.5	4.0	4.1	3.6	230
8	2.6	2.7	0.0	1.0	2.4	0.0	0.0	0.0	0.0	3.0	3.6	3.1	3.3	3.4	2.9	2.8	3.4	2.9	190
22F	1.6	4.1	1.9	0.0	2.4	0.0	1.6	0.0	0.0	0.6	0.9	0.8	2.2	4.3	4.1	3.3	3.3	2.9	187
9N	2.6	1.4	1.9	4.0	1.2	5.6	1.6	1.9	1.9	1.8	3.1	1.0	1.1	2.0	1.9	2.3	2.7	2.3	146
18C	3.6	2.7	0.0	2.0	1.2	0.0	1.6	0.0	5.6	1.8	2.7	1.0	3.3	2.4	2.2	1.8	2.7	2.2	144
11A	3.1	2.7	1.9	2.0	3.6	0.0	1.6	1.9	0.0	0.6	0.9	2.1	3.0	2.0	1.4	1.8	2.7	2.1	134
10A	1.6	1.4	5.7	3.0	2.4	3.7	4.8	0.0	1.9	1.2	3.6	0.8	2.2	1.4	1.4	1.8	2.0	1.9	121
12F	2.6	2.7	1.9	2.0	7.1	5.6	3.2	1.9	0.0	3.0	2.2	2.9	0.7	0.7	0.6	1.1	1.7	1.6	102
23A	0.5	1.4	1.9	0.0	0.0	1.9	1.6	0.0	1.9	1.2	1.3	1.0	0.4	0.7	0.8	1.1	1.7	1.1	74
24F	1.6	0.0	1.9	1.0	1.2	1.9	0.0	0.0	0.0	1.8	0.4	0.5	1.1	0.7	1.1	0.8	1.7	1.1	72
35F	1.0	0.0	1.9	1.0	0.0	1.9	0.0	1.9	0.0	1.2	0.0	0.5	0.0	0.5	0.6	0.8	1.9	1.0	64
15B	1.0	2.7	0.0	0.0	1.2	0.0	1.6	0.0	1.9	0.0	0.9	0.3	1.1	1.0	1.0	0.8	1.1	0.9	57
33F	1.6	1.4	1.9	1.0	0.0	0.0	1.6	1.9	0.0	0.6	0.0	1.0	1.5	1.0	0.3	0.6	0.9	0.8	51
20	1.0	2.7	3.8	0.0	0.0	0.0	0.0	0.0	0.0	0.0	0.0	0.8	0.7	1.0	0.5	0.6	0.4	0.6	38
38	0.0	0.0	1.9	0.0	0.0	1.9	0.0	0.0	0.0	0.6	0.4	0.8	0.7	0.2	0.5	0.3	1.1	0.6	38
5	1.6	0.0	1.9	1.0	1.2	0.0	0.0	0.0	0.0	0.6	0.9	0.3	0.0	0.2	0.6	0.5	0.7	0.6	36
17F	0.0	2.7	0.0	1.0	0.0	0.0	0.0	0.0	0.0	0.0	2.7	0.5	0.7	1.0	0.5	0.4	0.2	0.5	31
NT	0.0	0.0	0.0	0.0	1.2	0.0	0.0	0.0	0.0	0.6	0.4	0.0	0.0	0.5	0.5	0.7	0.5	0.5	30
15A	0.0	0.0	0.0	2.0	0.0	0.0	3.2	1.9	0.0	1.2	1.3	0.3	0.7	0.5	0.3	0.1	0.4	0.4	27
15C	1.6	0.0	0.0	2.0	0.0	1.9	0.0	0.0	1.9	0.0	0.4	0.5	0.4	0.2	0.6	0.5	0.1	0.4	27
16F	0.0	0.0	0.0	2.0	1.2	0.0	0.0	1.9	0.0	1.2	0.9	0.8	0.7	0.2	0.6	0.1	0.4	0.4	27
9A	0.5	1.4	1.9	1.0	2.4	0.0	1.6	0.0	0.0	3.0	2.2	0.5	0.0	0.7	0.0	0.2	0.0	0.4	26
31	1.0	0.0	1.9	1.0	0.0	0.0	0.0	0.0	0.0	1.2	0.9	0.8	0.0	0.2	0.5	0.1	0.5	0.4	25
33A	0.0	0.0	0.0	0.0	0.0	0.0	0.0	0.0	0.0	0.0	0.0	0.0	0.4	0.5	0.8	0.5	0.2	0.3	21
34	0.5	0.0	0.0	0.0	0.0	0.0	0.0	0.0	0.0	0.0	0.9	0.5	0.0	0.2	0.3	0.2	0.2	0.2	16
13	0.0	4.1	0.0	0.0	0.0	0.0	0.0	0.0	0.0	0.6	1.3	0.0	0.4	0.0	0.0	0.1	0.2	0.2	12
18A	0.0	0.0	1.9	0.0	0.0	0.0	0.0	0.0	0.0	0.0	0.0	0.5	0.0	0.3	0.2	0.2	0.1	0.2	12
2	0.5	0.0	1.9	1.0	0.0	1.9	0.0	1.9	0.0	0.0	0.0	0.0	0.0	0.0	0.0	0.4	0.0	0.2	12
9L	0.0	0.0	0.0	0.0	0.0	0.0	1.6	0.0	0.0	1.2	0.9	0.3	0.4	0.0	0.2	0.2	0.0	0.2	12
23B	0.0	0.0	0.0	0.0	0.0	0.0	0.0	0.0	0.0	1.2	0.0	0.0	0.4	0.2	0.2	0.1	0.2	0.2	11
rough	0.0	0.0	1.9	1.0	1.2	0.0	0.0	0.0	0.0	0.0	1.3	1.3	0.0	0.0	0.0	0.0	0.0	0.2	11
10B	0.0	0.0	0.0	0.0	0.0	0.0	1.6	0.0	0.0	0.0	0.0	0.0	0.4	0.3	0.0	0.2	0.2	0.2	10
18F	0.5	2.7	0.0	1.0	0.0	0.0	0.0	0.0	0.0	0.0	0.0	0.3	0.0	0.2	0.0	0.2	0.0	0.2	10
12A	0.0	0.0	0.0	0.0	0.0	0.0	0.0	0.0	0.0	0.0	0.4	0.0	0.0	0.0	0.5	0.2	0.1	0.1	9
28A	0.0	0.0	0.0	0.0	0.0	0.0	0.0	0.0	0.0	0.0	0.9	0.3	0.0	0.0	0.2	0.2	0.0	0.1	8
35B	0.0	0.0	0.0	0.0	0.0	0.0	0.0	0.0	0.0	0.0	0.0	0.0	0.0	0.0	0.0	0.2	0.2	0.1	8
15F	0.5	0.0	0.0	1.0	0.0	0.0	0.0	1.9	0.0	0.0	0.4	0.3	0.4	0.0	0.0	0.1	0.0	0.1	7
7C	0.5	0.0	0.0	1.0	0.0	0.0	0.0	0.0	0.0	0.0	0.0	0.3	0.0	0.2	0.0	0.0	0.2	0.1	7
11F	0.5	0.0	0.0	0.0	0.0	0.0	0.0	0.0	0.0	0.0	0.0	0.0	0.0	0.0	0.0	0.3	0.0	0.1	6
10F	0.5	0.0	0.0	0.0	0.0	1.9	0.0	0.0	0.0	0.0	0.0	0.3	0.0	0.0	0.0	0.1	0.1	0.1	5
12B	0.0	0.0	0.0	0.0	0.0	0.0	0.0	0.0	0.0	0.6	0.0	0.0	0.0	0.0	0.0	0.2	0.0	0.1	5
Others^⋆^	1.0	2.7	1.9	6.0	1.2	0.0	8.1	0.0	0.0	1.8	0.4	1.6	0.0	0.9	0.3	0.8	0.6	0.9	58
Total	100	100	100	100	100	100	100	100	100	100	100	100	100	100	100	100	100	100	6,446

Not sero- typed	57.5	82.8	82.8	69.6	68.4	71.3	70.5	77.1	73.4	61.4	47.7	30.7	31.8	2.3	0.0	0.1	0.0	29.3	2,671

NT: nontypeable; *n*: number of isolates tested.

Others^⋆^ includes the serotypes (number of isolates): 33B (4), 35A (4), 11B (3), 19C (3), 22A (3), 29 (3), 35C (3), 37 (3), 45 (3), 18B (2), 19B (2), 21 (2), 24A (2), 24B (2), 25F (2), 28F (2), 36 (2), 48 (2), 7B (2), 12 (1), 17A (1), 18 (1), 19 (1), 23 (1), 39 (1), 6 (1), 7A (1), 9 (1).

**Table 2:** Distribution of isolates serotyped among pneumococcal isolates sent to the NRCS in Germany (1992–2008).

Isolates serotyped	1992 (%)	1993 (%)	1994 (%)	1995 (%)	1996 (%)	1997 (%)	1998 (%)	1999 (%)	2000 (%)	2001 (%)	2002 (%)	2003 (%)	2004 (%)	2005 (%)	2006 (%)	2007 (%)	2008 (%)
*children*																	
serotyped (%)	92,2	82,9	96,6	65,4	38,7	92,9	97,6	96,8	96,2	100,0	99,6	99,2	100,0	100,0	100,0	100,0	100,0
serotyped (*n*)	47	29	28	17	12	158	161	182	200	239	258	244	265	316	287	273	232
total	51	35	29	26	31	170	165	188	208	239	259	246	265	316	287	273	232

*adults*																	
serotyped (%)	42,5	17,2	17,2	30,4	31,6	28,7	29,5	22,9	26,6	38,6	52,3	69,3	68,2	97,7	100,0	99,9	100,0
serotyped (*n*)	193	74	53	100	84	54	62	53	54	165	225	384	270	588	628	1654	1805
Total	454	429	309	329	266	188	210	231	203	428	430	554	396	602	628	1655	1805

*overall*																	
serotyped (%)	47,5	22,2	24,0	33,0	32,3	59,2	59,5	56,1	61,8	60,6	70,1	78,5	80,9	98,5	100,0	99,9	100,0
serotyped (*n*)	240	103	81	117	96	212	223	235	254	404	483	628	535	904	915	1927	2037
total	505	464	338	355	297	358	375	419	411	667	689	800	661	918	915	1928	2037
